# Transient Global Amnesia in a 60-year-old female with Post-traumatic Stress Disorder

**DOI:** 10.7759/cureus.5792

**Published:** 2019-09-28

**Authors:** Eduardo D Espiridion, Jayesh Gupta, Andre Bshara, Zachary Danssaert

**Affiliations:** 1 Psychiatry, Frederick Memorial Hospital, Frederick, USA; 2 Psychiatry, Drexel University College of Medicine, Philadelphia, USA; 3 Medicine, Drexel University College of Medicine, Philadelphia, USA; 4 Medicine, Philadelphia College of Osteopathic Medicine, Philadelphia, USA

**Keywords:** transient global amnesia, transient ischemic attack, traumatic brain injury, psychogenic amnesia, post traumatic stress disorder

## Abstract

This is a case report involving a 60-year-old female who developed transient global amnesia (TGA) after an emotional psychotherapy session in the framework of post-traumatic stress disorder (PTSD). She presented to the local emergency room, three days after her psychotherapist appointment, with complaints of memory impairment. She and her husband were worried about acute stroke since it was a sudden memory loss. The patient discussed with her psychotherapist a physical assault that occurred five years ago. Three days after that discussion, the patient developed the memory loss acutely. PTSD is associated with dissociative and retrograde amnesia. This case report demonstrates that PTSD can present with anterograde amnesia in the form of TGA.

## Introduction

Transient global amnesia (TGA) is a sudden impairment of memory characterized by anterograde amnesia that resolves gradually over the course of several hours. In this clinical syndrome, patients demonstrate repetitive questioning due to the inability to encode new memories [1]. This condition is seen mostly in individuals between the ages, 50 to 70 [2] and often resolves within 24 hours [1-2]. Diagnostic criteria include witness of an attack by a capable observer, presence of anterograde amnesia, no loss of personal identity, no neurological symptoms during or after an attack, absence of epileptic features, resolution of TGA within 24 hours, no recent head injury, and no history of seizures or active epilepsy [1]. 

## Case presentation

The patient is a 60-year-old married Caucasian female who presented in the emergency room of a local community hospital with a chief complaint of acute memory loss. She reported being confused for the past two days. She had no recollection about what has transpired the past two days but she remembered the details of her identity and that of her family. She could not recall the circumstances that brought her to the hospital, the name and dosages of her medications, her work history, or her medical history. The patient denied abusing any illicit drugs. She could not identify neighbors who visited her. She reported to her husband that she was not anxious about the memory loss. Her husband was worried that she was having a stroke, so they rushed to the hospital emergency room. Patient struggled to remember details of her morning routine, including her jogging at the neighborhood park, despite the husband's insistence that they ran together. Further inquiry revealed that the patient had attended a recent psychiatric appointment. She had an appointment with her outpatient psychotherapist three days before the acute onset of the memory loss. During that appointment, they discussed the person who physically assaulted her five years ago. She was a teacher in a special education residential program where one of her students with autism spectrum disorder punched her repeatedly on the head until she lost consciousness. Since the assault, the patient has been complaining of nightmares and flashbacks about the attack. She became socially withdrawn.

After the psychotherapist appointment, there was an exacerbation of the patient's anxiety symptoms leading up to the memory loss. There was no loss of consciousness during this period. During the psychiatric evaluation, the patient became tearful when discussing the details of the beating she endured. She described vivid memories of the assault as well as unpleasant dreams related to it. She gets particularly anxious whenever she sees a person that resembles her attacker. She could not perform the task of immediate retention and recall. The patient only identified one out of three words after five minutes. She could not complete her other cognitive examinations because she became tearful and requested to end it. The patient's husband reported that she was diagnosed with post-traumatic stress disorder (PTSD) three years ago and has since been treated with sertraline 100 mg per day. She is also being treated with cognitive-behavioral therapy.

This is reportedly the first time she has experienced memory loss. She denied any symptoms of depression, suicidal thoughts, mania, delusions, or hallucinations. There was no family psychiatric history. Mental status examination described a patient who looked appropriate to her stated age, dressed in a hospital gown, and was calm and cooperative with good eye contact during the psychiatric evaluation. There was no agitation observed. Her speech was coherent and goal-directed. She appeared anxious and restless; her mood was "stressed" and her affect was anxious. The patient was ambulating with good muscle strength. There was no aphasia or apraxia. Preliminary work-up showed a complete blood count with a white blood cell count of 5,900 cells per cubic millimeter of blood. Her basic metabolic panel showed serum sodium of 144 mEq/L. The urine toxicology screen was negative for illicit drugs. Magnetic resonance imaging (MRI) of the brain without contrast, magnetic resonance angiography of the circle of Willis, and a computed tomography (CT) scan of the head without contrast showed no acute intracranial pathology. After 48 hours, her cognitive symptoms improved. There was no anti-coagulation treatment. Her vital signs were stable and her 24-hour electroencephalogram monitoring showed no abnormalities. She was discharged home after 48 hours of observation with no cognitive problems.

## Discussion

The patient presented with a past medical history significant for PTSD which is being managed with sertraline and cognitive behavioral therapy. The memory loss started three days after she recounted the traumatic experience during a psychotherapy session. She presented with cognitive impairment with problems with her immediate retention and recall. Medical work-ups were negative so a psychiatric consultation was requested. TGA is a sudden impairment of memory characterized by anterograde amnesia that resolves gradually over the course of several hours. In this clinical syndrome, patients demonstrate repetitive questioning due to the inability to encode new memories [1]. This condition is seen mostly in individuals between the ages of 50 to 70 [2] and often resolves in 24 hours [1-2]. Diagnostic criteria include witness of attack by a capable observer, presence of anterograde amnesia, no loss of personal identity, no neurological symptoms during or after the attack, absence of epileptic features, resolution of TGA within 24 hours, no recent head injury, and no history of seizures or active epilepsy [1].

This case report is unique since there is no known report acknowledging the link between PTSD and TGA. There is also no report linking the emergence of TGA from psychotherapy treatment of PTSD. However, sources identify stress as a key precipitant of TGA [2-6]. The rate of stress-related TGA varies between 14% [4] and 50% [6] of the total TGA. This shows the close association between stress and TGA. Other reported causes include sexual intercourse, migraines, orgasms, emotional stress, swimming, sudden temperature change, and coughing [2-3,7]. There is a report of a TGA triggered by a nightmare [8] which has resemblance with our case report. These reports broaden possible etiologies to pre-existing traumatic conditions. Residual emotional trauma in PTSD may lead to TGA episodes. This was experienced as an intrusive image of an attacker by our patient. Acute flashback with subsequent emotional numbness and dissociative subtype of PTSD were considered in the differential diagnoses. This patient denied that the TGA event was a flashback of the traumatic event that she experienced and she denied any loss of personal identity. Dissociative type of PTSD is associated with both. While memory loss is seen in PTSD-related dissociative amnesia, the acute-onset anterograde memory loss makes this diagnosis less likely. TGA traditionally starts soon after a stimulus [9], but our patient's TGA began three days after a psychotherapy session when she recounted her traumatic experience. This was the only significant stimulus to have accounted for the TGA. Due to the nature of the disease and PTSD, we are unable to obtain immediately before the onset of the TGA. Our patient may have been re-experiencing the emotional trauma from her attack immediately before the amnesia began. Our patient's cognitive deficits resolved after 48 hours. This is different from the study that looked at 142 cases of TGA where the cognitive impairment symptoms lasted an average of 5.6 hours, and resolved within 24 hours [10]. This is a unique aspect of this case that does not fit the known criteria of TGA. TGA with two years of retrograde amnesia and which persisted for a week has been reported [3]. TGA may also present with mild lingering neuropsychiatric deficits after the 24-hour benchmark [11]. Trauma is classically associated with retrograde amnesia [12-13].

## Conclusions

There is a lack of documentation of TGA reported in individuals with a history of PTSD. We have presented a delayed-onset TGA resulting from PTSD which persisted beyond the 24-hour diagnostic standard. Though trauma has classically been associated with retrograde amnesia, this case demonstrates anterograde and identity memory impairments likely induced by her PTSD. It is also possible that this presentation may be labeled PTSD-related dissociative amnesia. Further documentation is needed to explore the relationship of TGA precipitated by emotional stress in PTSD.
